# Habitat filtering and exclusion of weak competitors jointly explain fern species assemblage along a light and water gradient

**DOI:** 10.1038/s41598-017-00429-9

**Published:** 2017-03-22

**Authors:** Hui Zhang, Shidan Zhu, Robert John, Ronghua Li, Hui Liu, Qing Ye

**Affiliations:** 1 Key Laboratory of Vegetation Restoration and Management of Degraded Ecosystems, South China Botanical Garden, Chinese Academy of Sciences, 723 Xingke Road, Tianhe District, Guangzhou 510650 P.R. China; 2 Guangdong Provincial Key Laboratory of Applied Botany, South China Botanical Garden, Chinese Academy of Sciences, 723 Xingke Road, Tianhe District, Guangzhou, 510650 China; 3 Department of Biological Sciences, Indian Institute of Science Education and Research, Kolkata, Mohanpur, West Bengal, 741246 India

## Abstract

Fern species are an important component of the diversity of forest plant communities, but very little is known about how fern communities assemble in different environments. In this study, we use multiple trait-based tests to examine the relationships between several key eco-physiological traits which are direct indicators of shade and drought tolerance, and the abundance of fern species in pine forest (PF), pine and mixed broad leaf forest (PMBF) and matured broad leaf forest (MBF) in southern China. These forests are characterized by decreasing light but increasing water availability during succession, and the fern communities correspondingly differ in species composition. We tested community assembly using functional trait distributions and found that habitat filtering and exclusion of weak competitive traits among coexisting species jointly explain fern shade tolerance as measured by photosynthetic capacity (PR), photosynthetic nutrient efficiency (PNUE and PPUE) and water use efficiency as measured by carbon isotope ratio (CIR), and constitute important determinants of fern community assembly in all three forests. These observed fern plant strategies are consistent with known responses of other plant taxa such as flowering plants in similar successional environments and illustrate the value of functional trait based analyses to study community assembly.

## Introduction

Identifying the mechanisms generating changes in species abundances in space and time is a central question in ecology^1^. In forests ecosystems, this is a question of practice as well as theoretical relevance, on account of swiftly advancing deforestation and predicted climate change^2^. The recent focus has been on using plant functional trait distributions^3^, among species to identify the mechanisms that determine species distributions and abundance in relation to the environment and biotic interactions. Since functional traits capture important aspects of species’ morphology, physiology, and life strategies, they reveal attributes such as abiotic tolerance and competitive ability that can determine plant fitness at a given site^1^.

In particular, trait dispersion patterns among species within communities have been used to draw inferences on two fundamental processes that determine plant species abundance at a site^4, 5^. Deterministic processes involve the role of habitat filtering, which selects for optimal trait values that confer appropriate abiotic physiological tolerance to a site, and thus lead to a convergence in trait distributions among species^4, 6, 7^. Deterministic effects may also entail strong competitive interactions among similar species, and may thus select for dissimilar trait values leading to divergence in trait distribution patterns^8, 9^. However, competitive interactions may also lead to trait convergence among species if traits that confer weak competitive ability were eliminated and only competitively equivalent species remain^10–12^. Stochastic processes invoke ecological drift as the main factor that determines species abundances, where trait differences are uncorrelated with species abundances and species are considered ecologically equivalent^13, 14^. Such species may coexist for long periods of time but are not expected to show significant convergence or divergence in functional trait dispersion patterns. Although such trait dispersion patterns have been widely used to infer the importance of deterministic and stochastic effects on plant species abundance, the confounding influences of abiotic and biotic trait convergence need to be resolved.

Our understanding of environmental and biotic control on plant species abundance has mostly come from studies on seed producing plants. However, comparisons across taxa are essential for identifying generalities on the importance of deterministic versus stochastic processes in structuring forest plant communities^15^. Our approach in this study here is to test how these two classes of processes affect a distinct group of understory plants, the ferns, which are different in their life-form and biology compared to the seed plants^15^. The influence of ferns on tree community dynamics is not well understood, but fern species can both inhibit and facilitate the establishment of seed plants. Fern species stands can facilitate the establishment of seed plants by slowing soil erosion and by providing establishment sites on their trunks for seed germination^16, 17^. Fern species can also inhibit establishment or growth of seed plants through altering light and soil nutrients due to competition for nutrients^18^ or light^19^. Although fern species represent a conspicuous fraction of tropical, temperate and cool wet forests^20^, to date, few studies have focused on this biological component^20–23^, compared to angiosperm tree species. Furthermore, our knowledge on deterministic and stochastic effects on tree communities may not be directly applicable to ferns, as the latter present two independent life phases: a tiny and relatively simple gametophyte and a well-developed, conspicuous sporophyte with different biotic and abiotic filters and ecological determinants of their performance^24^.

In this study, we test the effects of deterministic and stochastic processes on fern species community assembly in three types of forests: a Pine forest (PF), a Pine and Mixed Broadleaf Forest (PMBF) and a Mature Broadleaf Forest (MBF) in southern China. The PF and PMBF are successional stages of the MBF, and these stages are characterized by decreasing light but increasing water availability through succession. Given these strong differences between light and water availability in the forest understory, we expect to see very few fern species shared among these forest types. Our goals here are: i) to test whether traits that determine photosynthetic rate and drought tolerance influence species abundance in these successional communities; and ii) to quantify the roles of deterministic and stochastic effects on community assembly. We consider four key eco-physiological traits that capture fern shade-tolerance or photosynthetic capacity as well as drought stress tolerance. We therefore measured photosynthesis rate (PR), photosynthetic nitrogen use efficiency (PNUE), photosynthetic phosphorus use efficiency (PPUE), and water use efficiency, measured as carbon isotope ratio (CIR) (δ^13^C) of leaves. The selection of these four traits is based on the observations of decreased light but increased water availability in the understory environment from PF to MBF forest^25, 26^, which is expected to influence the physiology and function of fern species^22, 27–31^. We hypothesize that 1) abundant fern species in early successional communities tend to have high photosynthetic capacity (high PR) and high photosynthetic nutrient efficiency (high PPUE and PNUE), but may therefore suffer lower long-term water use efficiency, whereas the dominant fern species in late successional communities would have low photosynthetic capacity (low PR), low photosynthetic nutrient efficiency (low PPUE and PNUE) and high long-term water use efficiency, and 2) that the strong environmental effects of light and water availability would lead to trait convergence due to habitat filtering in all four traits.

## Materials and Methods

### Study site

Our study site is the species-rich sub-tropical forest located in Dinghushan Natural Forest Reserve (23°09′N, 112°33′E), Guangdong Province, southern China. The forest covers an area of 1,156 ha, with an elevation range from 14 m to 1000 m above sea level. The area is characterized by a typical sub-tropical monsoon climate with a mean annual air temperature of 21.4 °C, and mean annual precipitation of 1,956 mm with a bimodal distribution pattern between April-September and October-March. The average relative humidity is about 80%. The three forests, Pine forest (PF), pine and broadleaf mixed forest (PBMF), and monsoon evergreen broadleaf forest (MEBF) represent early-, mid- and late- successional stages, respectively, of the typical monsoon evergreen broadleaf forests in Dinghushan. The PF forest, which is approximately 22 ha in area and occupies the periphery of the reserve was initially planted in 1950s with a single pine species, *Pinus massoniana* (D. Don), The PMBF forest, which is approximately 557 ha in area and had developed from an earlier established PF forest is located between the central area and periphery of the reserve. The MBF forest, approximately 218 ha in area is located in the central part of the reserve. The characteristics of the experimental sites are shown in Table 1.

**Table 1:** The environmental characteristics of each successional forest community.

Forest type	stand age (year)	Elevation (m)	Slope	Mean air temperature (°C)	Relative humidity (%)	Annual litter fall Mass (Mg ha^−1^)	Soil water storage (0–75 cm) (mm)	Cannopy height (m)	Cannopy coverage (%)	Average PPFD during a day (μmol m^−2^ s^−1^)
PF	about 60	130–200	10°–20°	22.7	80	2.53	254.36	about 8	about 40–50	About 471
PMBF	about 110	150–220	10°–25°	20.9	82	7.31	324.98	about 12	about 70–80	About 280
MBF	about 400	160–230	15°–20°	20.4	87	8.84	381.03	about 15	>95	About 65

Pine forest (PF), pine and mixed broadleaf forest (PMBF), and matured broadleaf forest (MBF), as cited from Li and Zhu *et al.* ^27^.

### Measurement of species composition and abundance of ferns

We tallied all fern species in each forest type and compared the species composition. Species abundance can be measured by the number of individuals, or by biomass or resource use^32^. In this analysis, we chose biomass because it was the appropriate measure for scaling from plant traits to community assembly processes^33, 34^. Twenty 10 × 10 m^2^ quadrats were regularly arranged in six parallel transects, with 20 m intervals between adjacent quadrats. In each quadrat, the aboveground parts for each fern species were clipped and taken to the laboratory, where samples were oven-dried at 100 °C for 2 days and then weighed to determine the aboveground biomass for each species to estimate species abundance. Species abundance for fern species at each successional stage was quantified as the total above ground biomass of each species found in the twenty 10 × 10 m^2^ quadrats.

### Collection of functional traits

We quantified plant shade-tolerance via photosynthesis rate, photosynthetic nitrogen and phosphorus efficiency. We also quantified leaf carbon isotope ratio, which was closely related to plant long-term water use efficiency. Importantly, we measured traits of the same fern species in each successional forest separately to ensure that intra-specific variation was appropriately incorporated into our analyses.

### Leaf nutrient and phosphorus concentration

We collected 40 fully expanded sun-exposed leaves each individual and oven dried them for 72 h at 70 °C to determine their dry weight. The dried leaves were then ground and homogenized for subsequent analyses. Leaf chemical analysis was conducted in the Public Laboratory of South China Botanical Garden. Total nitrogen concentration (N) was determined by Kjeldhal analysis and total phosphorus concentration (P) using atomic absorption spectrophotometry.

### Photosynthesis rate, photosynthetic nitrogen and phosphorus use efficiency

Maximum photosynthetic rate (*PR*) was measured between 9:00–12:00 with a portable photosynthesis system (Li-6400, LiCor, Lincoln, Nebraska, USA). The PPFD was set at 1000 μmol m^−2^ s^−1^ to ensure that light-saturated photosynthetic rates were measured for all species selected in this study (Zhang *et al.*, unpublished data). Ambient CO_2_ concentration and air temperature were maintained at 390 μmol mol^−1^ and 28 °C, respectively. Ten individual plants for each fern species were randomly selected and at least 10 leaves of each species were used for the photosynthetic measurements. Photosynthetic nitrogen use efficiency (PNUE) and photosynthetic phosphorus use efficiency (PPUE) are defined as the ratio of PR to leaf N content and P content respectively.

### Leaf carbon isotope ratio

Carbon isotope ratio (CIR), calculated as δ^13^C (per mil), can be used to estimate long-term water-use efficiency of leaves in natural vegetation^26^. The CIR was measured with an elemental analyzer (Flash EA 1112, Thermo Electron Corporation, Waltham, MA, USA) interfaced to an isotope ratio mass spectrometer (Thermo Finnigan MAT DELTAplusXP, Thermo Electron Corporation, MA, USA) at the Institute of Desertification Studies, Chinese Academy of Forestry (Beijing, China).

### Statistical methods

#### Species abundance versus trait relationships along succession

We log-transformed all measured functional traits and the values of species abundance to normalize the data. Then we performed parametric (Pearson’s) correlation analyses of the relationships between functional traits and fern species abundance in the three forests.

#### Trait dispersion patterns and community assembly

We analyzed observed trait dispersion patterns by comparing the observed functional diversity (FD) with the FD of null communities derived using two approaches, (i) where randomizations of abundance and trait values were carried out within each forest type, and (ii) where randomizations were carried out by drawing species and trait values across all forest types (the species pool). Using this combination of approaches, we could resolve the confounding effects of habitat filtering and exclusion of weak competitive traits (see the Introduction section above) on any observed trait convergence patterns within communities.

(i) Randomization tests within each forest type: Functional diversity of communities was analyzed for each trait separately. To obtain functional diversity we computed the mean pair distance (MPD), which is the mean dissimilarity among all possible pairs of species within a community, weighted by species abundances^35^. Although other functional diversity indices are available, we chose MPD because the selected traits (PR, PNUE, PPUE and CIR) are recognized to represent orthogonal axes of plant strategies^36, 37^ and this FD measure is also relatively independent of species richness^38^. We used the “MPD” function implemented in the ‘picante’ library (http://cc.oulu.fi/?jarioksa/softhelp/picante.html) in the R software (R Development Core Team 2017) to calculate FD for PR, PNUE, PPUE and CIR.
  We checked for significant convergence or divergence in FD with respect to an ensemble of null communities, which was based on the expectation that species and trait values were randomly distributed within each forest community. Randomization tests were applied to calculate “null” distributions of both species composition (i.e., on the species × quadrat matrix) and functional diversity^39^. For this method, we used the “randomizeMatrix” function implemented in the ‘picante’ library (http://cc.oulu.fi/?jarioksa/softhelp/picante.html) in the R software (R Development Core Team 2017). Reshuffling the species × quadrat matrices was done with three constraints, i.e. while keeping 1) the same number of species (species richness) per plot in the permuted and observed data, 2) the same number of total species occurrences per region (i.e. number of plots where the species occur in a region), and 3) the total abundance of species in a region constant (i.e., the sum of the number of quadrats occupied in all plots). We therefore compared the observed FD (FDcom) within communities to the FD simulated in 1000 randomly assembled communities (FDnull), using the standard effect size index (SES)^40^:
  1 $${\rm{SES}}=\frac{{\rm{FDcom}}-{\rm{FDnull}}}{{\rm{FDsd}}}$$
  Here FDsd represents the standard deviation of FD generated from null communities. Values of SES > 0 suggest the prevalence of trait divergence, while SES < 0 suggests trait convergence, and SES ~ 0 suggests the prevalence of random trait distributions. Since this randomization involves only species from within the community, the observed trait convergence must result from both habitat filtering and exclusion of weak competitive traits, while trait divergence must primarily reflect exclusion of similar species.
(ii) Randomization using the species pool: deBello *et al.* ^38^ proposed an operational framework in which the FD within communities (FDcom) is compared to the corresponding FD for a given species pool (FDpool)^38^, where both measures are standardized by the number of the species in the community. FDpool includes the set of species that could potentially occupy a site in the absence of environmental and dispersal limitation filters. By comparing these FD patterns one can therefore infer biotic processes, *i.e.*, trait divergence (FDcom/FDpool > 1) indicating strong niche differentiation or facilitation induced trait dissimilarity, trait convergence (FDcom/FDpool < 1) indicating exclusion of weak competitive traits causing increased trait similarity among coexisting species, and finally random community assembly (FDcom/FDpool ~ 1). We defined the species pool as all species that occur in all of the sixty 10 × 10 m^2^ sampling quadrats in the three successional forest types. The MPD for each functional trait (PR, PNUE, PPUE and CIR) of all species found in all of the three successional forest communities was calculated to represent FDpool for each trait. Then FDcom for each functional trait (MPD) was also calculated for each successional forest community separately, and the values of the FDcom/FDpool ratio were compared.

## Results

We recorded the highest species richness of 7 fern species in Mature Broadleaf Forest and Pine Mixed Broadleaf Forest, with marginally lower species numbers of 5 fern species in Pine Forest. The species list and growth form of each species are given in the Table 2. We found that very few species were shared among the three forest types, with only 2 species shared between PF and PMBF, no species shared between PF and MBF, and 2 species shared between PMBF and MBF. Therefore, no fern species were common to all three forests.

**Table 2:** 19 fern species found in pine forest (PF), pine and mixed broad leaf forest (PMBF) and matured broad leaf forest (MBF).

Family	Species	Distribution
Blechnaceae	*Blechnum orientale* L.	PF
Thelypteridaceae	*Cyclosorus parasiticus* (L.) Farewell.	PF
Gleicheniaceae	*Dicranopteris dichotoma* (Thunb.) Bernh.	PF
Nephrolepidaceae	*Nephrolepis auriculata* (L.) Trimen	PF
Lindsaeaceae	*Schizoloma ensifolium* (SW.) J. Sm.	PF
Lindsaeaceae	*Schizoloma heterophyllum* (Dryand.) J. Sm.	PMBF
Lygodiaceae	*Lygodium japonicum* (Thunb.) SW.	PMBF
Lindsaeaceae	*Schizoloma ensifolium* (SW.) J. Sm.	PMBF
Dicksoniaceae	*Cibotium barometz* (L.) J. Sm.	PMBF
Adiantaceae	*Adiantum flabellulatum* L. Sp	PMBF
Gleicheniaceae	*Dicranopteris dichotoma* (Thunb.) Bernh.	PMBF
Aspidiaceae	*Hemigramma decurrens* (Hook.) Cop.	PMBF
Cyatheaceae	*Alsophila podophylla* Hook.	MEF
Angiopteridaceae	*Angiopteris fokiensis* Hieron.	MEF
Dryopteridaceae	*Arachniodes exilis* (Hance) Ching	MEF
Dicksoniaceae	*Cibotium barometz* (L.) J. Sm.	MEF
Polypodiaceae	*Microsorum fortunei* (T. Moore) Ching	MEF
Thelypteridaceae	*Pronephrium triphyllum* (Sw.) Holtt.	MEF
Gleicheniaceae	*Dicranopteris dichotoma* (Thunb.) Bernh.	MEF

**Figure 1:**
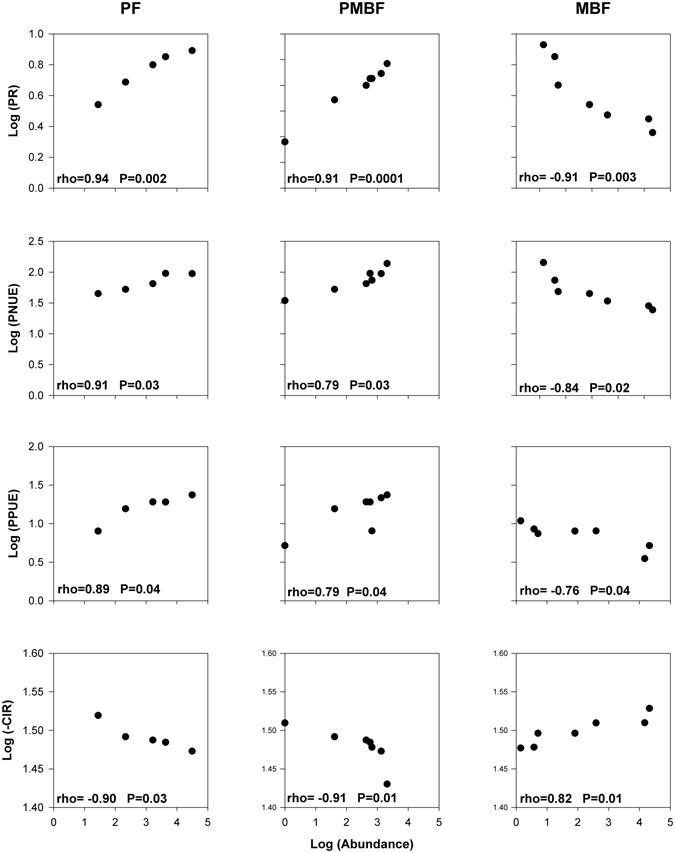
Spearman correlation analyses of the relationship between functional traits (photosynthesis rate (PR), photosynthetic nitrogen use efficiency (PNUE), photosynthetic phosphorus use efficiency (PPUE) and carbon isotope ratio (CIR)) and abundance of fern species in three forest successional stages (Pine forest (PF), pine and mixed broadleaf forest (PMBF), and matured broadleaf forest (MBF)). Panels illustrate the scatter of species’ abundance and functional traits (PR, PNUE, PPUE and CIR) of each species (filled points) in each forest successional stage. Pearson’s coefficients (rho) and p-value (P) are also shown.

We found statistically significant positive correlations between species abundance and traits for PR, PNUE and PPUE, but significant negative correlations between abundance and trait values for CIR of fern species in PF and PMBF forest (Fig. 1). In contrast, abundance of fern species in MBF were significantly negatively related to PR, PNUE and PPUE, but significantly positively related to CIR (Fig. 1).

**Figure 2:**
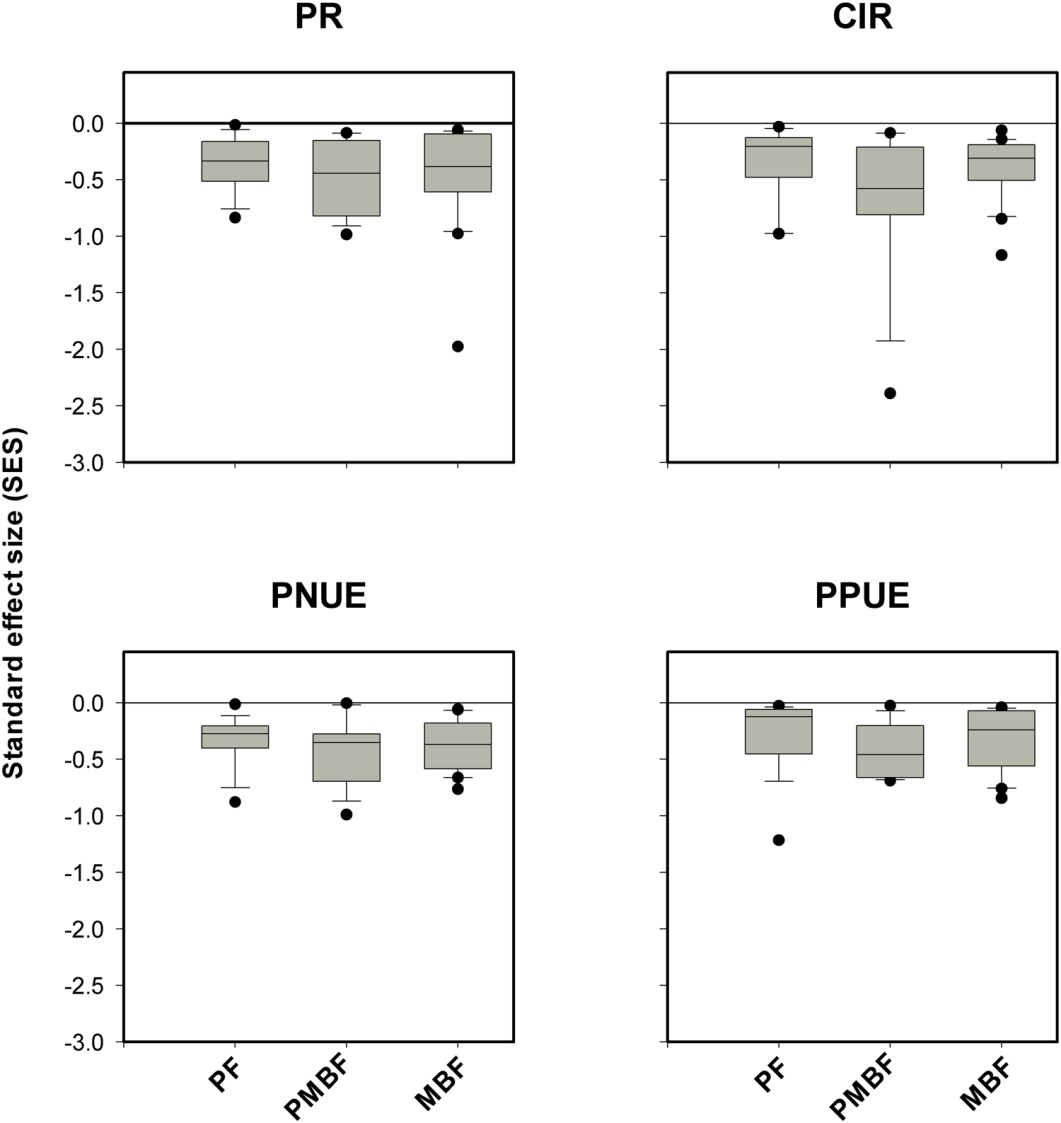
Values of standard effect size (SES) in three forest successional stages (Pine forest (PF), pine and mixed broadleaf forest (PMBF), and matured broadleaf forest (MBF)), obtained using 1000 communities simulated under a null model. SES > 0 represents trait divergence, SES < 0 indicates trait convergence and SES = 0 implies neither trait convergence nor divergence. The solid line inside the box represents the median of the data. The bottom and top of the box represent the 1st quartile and 3rd quartile of the data, respectively. The vertical lines extending from the box represent the data inside a range of 1.5 times the interquartile range from the box. An outlier is denoted by filled points.

We tested FD patterns for the four traits (PR, PPUE, PNUE and CIR) to detect convergence or divergence against expectations derived from a null model with no abiotic or biotic effects. We found evidence for significant trait convergence (SES < 0) in FD for all four traits, PR, PNUE, PPUE and CIR, in all three forests (Fig. 2). Furthermore, there was no trend in the values of SES from early to late successional communities for any of the four traits we studied (Fig. 2).

**Figure 3:**
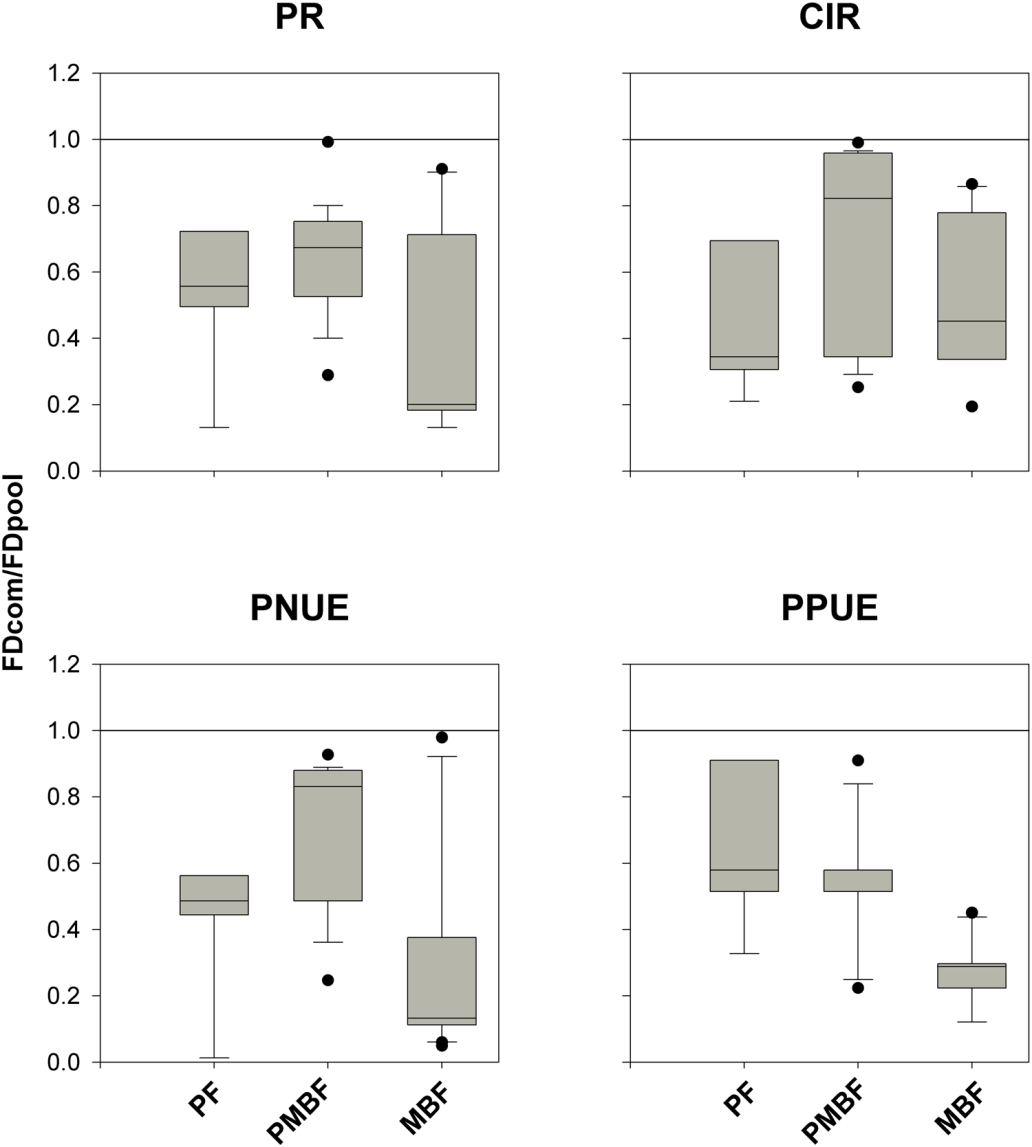
A boxplot showing the distributions of values of FDcom/FDpool ratio in three forest successional stages (Pine forest (PF), pine and mixed broadleaf forest (PMBF), and matured broadleaf forest (MBF)). FDcom/FDpool > 1 represents strong competition exclusion, FDcom/FDpool < 1 indicates exclusion of weak competitors, and FDcom/FDpool = 1 implies random biotic interaction. The solid line inside the box represents the median of the data. The bottom and top of the box represent the 1st quartile and 3rd quartile of the data, respectively. The vertical lines extending from the box represent the data inside a range of 1.5 times the interquartile range from the box. An outlier is denoted by filled points.

Finally, we compared the observed FD with the FD of the species pool, the latter being indicative of expected FD if habitat filtering and dispersal limitation was already accounted for, and trait dispersion would only be due to biotic interactions (exclusion of weak competitive traits, or strong competitors or facilitation). We found evidence for exclusion of weak competitive traits, with FDcom/FDpool ratios being less than 1 for PR, PNUE, PPUE and CIR in all three successional communities (Fig. 3). We did not find any consistent trends in the FDcom/FDpool ratios for any of the traits except for PPUE, where the MBF showed strong convergence (Fig. 3).

## Discussion

We found directional shifts in the relationships between fern species abundance and four functional traits (PR, PNUE, PPUE and CIR) along the light and water gradient that is present in three successional forests. As we hypothesized, high light, but low water availability in early successional stages allow species with high photosynthetic capacity (high PR) and high photosynthetic nutrient efficiency (high PPUE and PNUE), but low long-term water use efficiency (high CIR) to dominate. By contrast, low light, but high water availability drive late successional communities to be dominated by species having low photosynthetic capacity (low PR), low photosynthetic nutrient efficiency (low PPUE and PNUE) and high long-term water use efficiency (low CIR). Our results demonstrate that deterministic processes (habitat filtering and exclusion of weak competitors) have a dominant role in fern community assembly in all three forests, with a presumably small influence of stochastic processes.

### The relationships between functional traits (PR, PNUE, PPUE and CIR) and abundance of fern species along light and water gradient

Photosynthetic rate affects a plant’s energy balance and a high rate is typically linked to a fast growth rate^27, 41^. Early colonizers are most likely fast-growing species requiring high photosynthetic capacity and nutrient use efficiency that might be responsible for their quick colonization^42, 43^. Our results show significant positive correlations between abundance and energy/resource acquisition traits (PR, PNUE and PPUE) in PF and PMBF (Fig. 1), consistent with the well-known pattern that species that are abundant in early successional forests are fast growing, a strategy that would help in colonizing new habitats^42–45^. At more advanced stages of succession, the amount of light reaching the ground is expected to decline. Fern species in PF and PMBF forests most likely experience higher light levels than those found in the MBF forest on account of the lower canopy height and cover (Table 1). Expectedly, resource competition and the need for greater investment in plant defense would not favor fast-growth strategies in late successional communities^3^. We found in this study a significant shift in the relationship between traits (PR, PNUE and PPUE) and fern species abundance from positive (in PF and PMBF) to negative (MBF) (Figs 1 and S1), indicating the shift in fern species growth strategies during succession^3^.

Drought stress, indicated by a decrease in the number of rainy days and diminished soil water content has been implicated in the directional changes in species composition and community structure in this subtropical forest^46^. Fern species in PF and PMBF forests arguably experience a consistently drier environment than those found in MBF forest on account of higher temperatures and lower soil water content (Table 1), thereby selecting for drought stress tolerance in early successional ferns. Leaf carbon isotope ratio can reflect this plant drought stress tolerance, and indeed, we found statistically negative correlations between abundance and CIR of fern species in PF and PMBF forest (Fig. 1). From a physiological standpoint, higher photosynthetic rates are generally associated with lower water use efficiency (WUE) due to higher stomatal conductance and the resultant higher transpiration rates^30, 31^. This inevitable tradeoff between photosynthesis and WUE imposes greater water stress in an already water-stressed early-successional environment. Therefore, drought tolerance is likely to be strongly selected in fern species of early successional forests, and our results are consistent with these observations on plant strategies.

In late successional habitats such as the MBF, which have relatively higher soil water content, WUE may not be under strong selection. Although late successional fern species may display higher long-term water use efficiency, this is likely due to lower photosynthesis rates, rather than any selection for WUE. Indeed, we did find that the correlation of fern abundance versus CIR shifted from significantly negative in PF and PMBF to significantly positive correlation in MBF (Fig. 1). Although drought stress tolerance and plant successional status has been widely studied in woody seed plants, the mechanisms largely pertain to stem hydraulic conductance^28–31^ and are therefore not relevant to drought tolerance in fern species. That is because fern species are mostly herbaceous or do not possess the elaborate vasculature of seed plants in case of tree forms. However it is important to note that since our assessment of fern drought tolerance is based on a leaf CIR, our inference on the role of photosynthesis in influencing WUE of fern is limited and deserves further investigation. Importantly, our results clearly show that fern species differ strongly in their responses to different light and water environment and are not ecologically equivalent^3^. Our findings also highlight the use of eco-physiological traits in differentiating the relative contributions of stochastic (ecological equivalence) and deterministic processes (niche differentiation) to community assembly in successional environments.

### Trait dispersion patterns of fern communities along light and water gradients

Strong convergence or divergence in functional trait diversity compared to expectations from given null models have been widely employed to study species coexistence^4, 7, 47–50^. It is argued that convergence or divergence in functional diversity arise mainly due to habitat filtering and strong competitive exclusion, respectively. Our results show strong convergence (SES < 0) in all of the four traits (PR, PNUE, PPUE and CIR) in PF, PMBF and MBF forests (Fig. 2), highlighting the important roles of habitat filtering in determining these four traits. However, it is important to note that^38^ comparable trait convergence patterns can also be obtained due to exclusion of weak competitive traits^38^. We resolved these separate contributions by comparing the differences between the observed FD (FDcom) and that generated from species pool (FDpool) and found evidence for the exclusion of weak competitive traits that caused the FDcom/FDpool ratio to show values that were consistently less than 1 in all three types of forest (Fig. 3). Together, the results from comparing FD patterns using SES and FDcom/FDpool ratios suggest that both habitat filtering and exclusion of weak competitive traits contribute to the observed strong trait convergence in PR, PNUE, PPUE and CIR in all three forests.

Observations on species distributions had already shown that the abundant fern species in early the successional pine forest do not survive in the late successional mature broad leave forest community. Our results shed light on the plant strategies with respect to resource acquisition and nutrient use efficiency that mediate these distributional patterns of fern species in these three forests^12^.

## Conclusions

Habitat filtering and competitive interactions jointly explain shifts in fern species strategies from higher photosynthetic capacity, greater photosynthetic nutrient use efficiency, but lower water use efficiency in a early successional forest to lower photosynthetic capacity and nutrient use efficiency, but higher water use efficiency in the late successional broadleaf forest. These responses could be due to decreasing light availability and decreasing drought stress with succession, but other biophysical factors could also play important roles. These observed plant strategies are consistent with the expected changes in the biophysical environment during succession, and are also consistent with responses known from other plant taxa, primarily flowering plants. These findings indicate that similar selective forces shape comparable plant strategies across different plant groups. Further, our integrated methods offer a promising approach to resolve confounding contributions of some deterministic processes (habitat filtering and biotic interactions), but more broadly the relative importance of deterministic and stochastic processes on plant community assembly.
